# Lack of Association between Past *Helicobacter pylori* Infection and Diabetes: A Two-Cohort Study

**DOI:** 10.3390/nu11081874

**Published:** 2019-08-12

**Authors:** Jeung Hui Pyo, Hyuk Lee, Sung Chul Choi, Soo Jin Cho, Yoon-Ho Choi, Yang Won Min, Byung-Hoon Min, Jun Haeng Lee, Heejin Yoo, Kyunga Kim, Jae J. Kim

**Affiliations:** 1 Center for Health Promotion, Samsung Medical Center, Sungkyunkwan University School of Medicine, 81 Irwon-ro, Gangnam-gu, Seoul 06351, Korea; 2 Department of Medicine, Samsung Medical Center, Sungkyunkwan University School of Medicine, 81 Irwon-ro, Gangnam-gu, Seoul 06351, Korea; 3 Statistics and Data Center, Research Institute for Future Medicine, Samsung Medical Center, 81 Irwon-ro, Gangnam-gu, Seoul 06351, Korea

**Keywords:** *Helicobacter pylori*, Diabetes Mellitus, impaired glucose tolerance, diabetic nephropathy, poor glycemic control

## Abstract

*Helicobacter pylori* (*H. pylori*) may be involved in diabetes and other insulin-related processes. This study aimed to investigate the associations between *H. pylori* infection and the risks of type 2 diabetes, impaired glucose tolerance (IGT), diabetic nephropathy, and poor glycemic control. We retrospectively evaluated 16,091 subjects without diabetes at baseline who underwent repeated health examinations. Subjects were categorized according to whether they were seropositive and seronegative for *H. pylori* infection. Hazard ratios (HRs) and 95% confidence intervals (CIs) were calculated using Cox proportional hazard models. The serological results were validated using an independent cohort (*n* = 42,351) based on a histological diagnosis of *H. pylori* infection. During 108,614 person-years of follow-up, 1338 subjects (8.3%) developed newly diagnosed diabetes, although the cumulative incidence of diabetes was not significantly related to serological *H. pylori* status. The multivariate Cox proportional-hazards regression models revealed that *H. pylori* seropositivity was not significantly associated with diabetes (HR: 1.01, 95% CI: 0.88–1.16; *p* = 0.854), IGT (HR: 0.98, 95% CI: 0.93–1.04; *p* = 0.566), diabetic nephropathy (HR: 0.99, 95% CI: 0.82–1.21; *p* = 0.952), or poor glycemic control (HR: 1.05, 95% CI: 0.90–1.22; *p* = 0.535). Similarly, histopathological findings of *H. pylori* infection were not significantly associated with diabetes (*p* = 0.311), diabetic nephropathy (*p* = 0.888), or poor glycemic control (*p* = 0.989). The findings from these large Korean cohorts indicate that there does not appear to be a role for past *H. pylori* infection in the development of diabetes, IGT, diabetic nephropathy, or poor glycemic control.

## 1. Introduction

*Helicobacter pylori* (*H. pylori*) plays a major pathogenic role in gastrointestinal diseases, including chronic gastritis, peptic ulcer disease, gastric cancer, and mucosa-associated lymphoid tissue lymphoma [1,2,3]. Recent studies have indicated that *H. pylori* infection may interfere with many biological processes and determine or influence the occurrence of many extra-gastric diseases, including hematologic, metabolic, cardiovascular, neurodegenerative, and allergic diseases [4,5,6,7,8]. Recent epidemiological and clinical studies have also suggested that chronic *H. pylori* infection may be associated with atherosclerotic disease, cardiovascular disease, and other metabolic syndromes [6,7,9,10,11].

Insulin resistance is a pathophysiological factor in these clinical conditions, and several studies have suggested that chronic *H. pylori* infection might impair insulin sensitivity [12,13]. Other studies have indicated that *H. pylori* infection may be related to the development and progression of type 2 diabetes mellitus (DM), as well as its complications [9,13,14,15,16,17,18,19,20]. However, other studies, including a recent meta-analysis, have failed to indicate that *H. pylori* infection was associated with the development or worsening of type 2 DM control [21,22,23,24]. This lack of consensus and the variable findings may be related to differences in the diagnosis of DM, bacterial detection, and confounding variables that were considered in the analyses. Therefore, this large two-cohort study aimed to investigate the association between *H. pylori* infection and the risks of DM, diabetic complications, and poor glycemic control.

## 2. Materials and Methods

### 2.1. Study Cohorts

**Figure 1:**
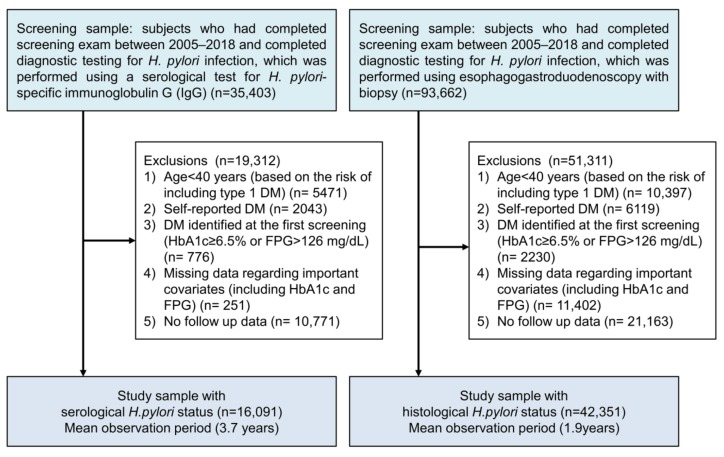
Flow diagram of study participants (*H. pylori*, Helicobacter pylori; DM, diabetes mellitus; HbA1c, glycated hemoglobin; FPG, fasting plasma glucose).

This retrospective study evaluated subjects who had completed at least two screening exams during 2005–2018 at the Center for Health Promotion, Samsung Medical Center. Regular health check-ups are very common in Korea because of the Industrial Safety and Health Law, and the National Cancer Screening Program recommends biennial health examinations, which include screening for several cancers [25]. The present study evaluated two separate cohorts; subjects who enrolled in the health check-up program, and subjects who completed diagnostic testing for *H. pylori* infection, which was performed using a serological test for *H. pylori*-specific immunoglobulin G (IgG) (*n* = 16,091) or esophagogastroduodenoscopy with biopsy (*n* = 42,351). We excluded subjects who were <40 years old (based on the risk of including type 1 DM), patients with self-reported DM, patients with DM identified at the first screening (glycated hemoglobin [HbA1c] of ≥6.5% or fasting plasma glucose [FPG] of >126 mg/dL), or patients with missing data regarding important covariates (including HbA1c and FPG), from each cohorts (Figure 1).

### 2.2. Data Collection

The comprehensive health-screening program included a questionnaire regarding lifestyle factors, medication use, and chronic disease; physical examinations; and a series of laboratory, radiologic, and endoscopic tests. Some subjects voluntarily paid for their health check-ups while others were partly supported by affiliated companies. This study’s retrospective protocol was approved by the institutional review board of Samsung Medical Center, and the study was conducted in accordance with the Declaration of Helsinki. The requirement for informed consent was waived because of the study’s retrospective nature. Data was extracted from the Clinical Data Warehouse Darwin-C of Samsung Medical Center for this study.

Anthropometric data were collected during common physical examinations by trained staff. The standardized questionnaire included information regarding personal medical history, medication use, alcohol consumption, smoking status, and physical activity. Heavy drinking was defined as ethanol consumption of ≥20 g/day. Regular exercise was defined as exercising ≥3 times per week with moderate intensity. Blood samples were obtained from the antecubital vein after the subject had fasted for 12 h. Serum blood tests were generally performed using common enzymatic methods with an automated analyzer (Hitachi Ltd., Tokyo, Japan), although HbA1c levels were measured using high-performance liquid chromatography with a Tosoh Glycohemoglobin Analyzer (Tosoh Bioscience Inc., Tokyo, Japan). Urinary albumin and creatinine concentrations were determined using an early morning spot urine sample. The estimated glomerular filtration rate (eGFR) was calculated using the Korean Society of Nephrology equation: eGFR = 186 × creatinine^−1.154^ × age^−0.203^ (mL/min/1.73 m^2^). For women, the eGFR was multiplied by a correction factor of 0.742.

### 2.3. Diagnosis of H. pylori Infection

The diagnosis of *H. pylori* infection was based on serological or histological findings. The *H. pylori*-specific IgG titers were measured using a commercially available enzyme-linked immunosorbent assay according to the manufacturer’s instruction (Bio-Rad Laboratories Inc., Hercules, California, USA). The histological *H. pylori* status was determined using hematoxylin and eosin staining of biopsy specimens that were obtained from suspicious lesions or for the diagnosis of *H. pylori* infection during esophagogastroduodenoscopy.

### 2.4. Diagnoses of DM and Diabetic Nephropathy

Based on the Korean Diabetes Association and American Diabetes Association definitions [26,27], we identified cases of DM (FPG level of ≥126 mg/dL or a HbA1c level of ≥6.5%), cases of IGT (FPG level of 100–125 mg/dL or a HbA1c level of 5.7–6.4%), and cases of poor glycemic control (a single HbA1c level of >6.5% in patients with DM). In patients with DM, diabetic nephropathy was identified based on albuminuria (serum creatinine of >30 mg/g) or a decreased eGFR (<60 mL/min/1.73 m^2^) [28].

### 2.5. Statistical Analysis

All statistical analyses were performed using SAS software (version 9.4; SAS Institute Inc., Cary, NC, USA) and R software (version 3.4.3; R Foundation, Vienna, Austria). Continuous variables were expressed as mean ± standard deviation, while categorical variables were expressed as number (%). Groups were compared using the Wilcoxon rank sum test and the chi-squared test. Cumulative incidences were calculated using the Kaplan-Meier method. Risk factors were identified using Cox proportional hazard regression models. Multicollinearity was checked using the variance inflation factor, although no variables had a result of >4. Hazard ratios (HRs) with 95% confidence intervals (CIs) were calculated for developing the various outcomes. Differences were considered statistically significant at *p*-values of <0.05.

Potentially confounding baseline factors were incorporated into three models. Model 1 was adjusted for age and sex. Model 2 was adjusted for the variables in Model 1 plus body mass index (BMI), systolic blood pressure, diastolic blood pressure, alcohol intake, smoking, and physical activity. Model 3 was adjusted for the variables in Model 2 plus levels of triglycerides, high-density lipoprotein (HDL)-cholesterol, and low-density lipoprotein (LDL)-cholesterol. The associations between *H. pylori* status and the various outcomes were initially evaluated using the serological data, and then validated using the histological data from the second cohort.

## 3. Results

### 3.1. Baseline Characteristics

The 16,091 eligible subjects included 9690 men (60.2%) and 6401 women (39.8%), with a mean age of 51.3 ± 7.5 years. Serological testing confirmed *H. pylori* positivity in 58.4% of the subjects, with the positive cases tending to be older, male, and not current smokers. No significant differences were observed according to *H. pylori* status in the values for systolic blood pressure, BMI, triglycerides, HbA1c, insulin, or urinary microalbumin. A positive serological result for *H. pylori* was associated with high but normal levels of total cholesterol and LDL-cholesterol, low but normal levels of HDL-cholesterol. In addition, *H. pylori* positivity was associated with low but normal values for FPG, C-peptide, and eGFR (Table 1).

### 3.2. Incidences of DM, IGT, and Diabetic Nephropathy

**Figure 2:**
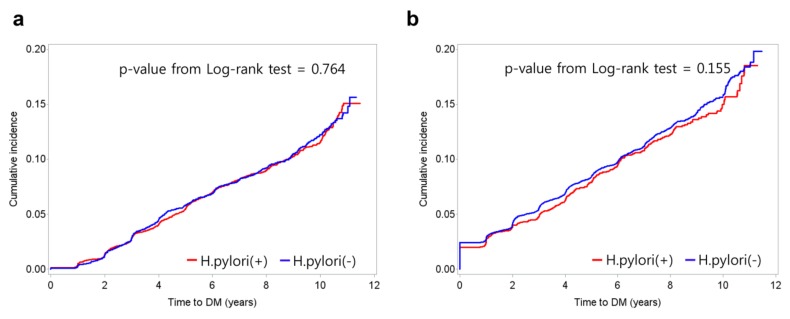
Cumulative incidence of diabetes according to H. pylori status based on serological findings (**a**) and histological findings (**b**).

During the 108,614 person-years of follow-up, 1,338 patients (8.3%) developed DM. No significant differences in the incidences of DM were observed according to *H. pylori* status based on serological findings (log-rank *p* = 0.764) and histological findings (log-rank *p* = 0.155) (Figure 2).

**Figure 3:**
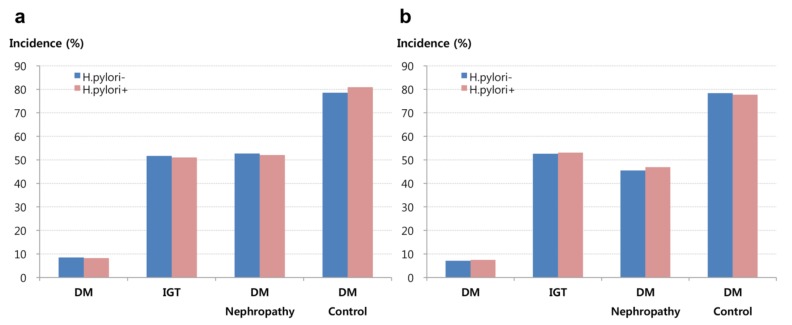
Incidences of diabetes, impaired glucose tolerance, diabetic nephropathy, and glycemic control according to *H. pylori* status based on serological findings (**a**) and histological findings (**b**). IGT, impaired glucose tolerance.

In cases with serological testing, the incidences of DM were 8.5% for *H. pylori*-negative cases and 8.2% for *H. pylori*-positive cases (log-rank *p* = 0.491). Similarly, there was no significant difference in the incidences of IGT between the *H. pylori*-positive and *H. pylori*-negative cases (51.6% vs. 51.0%, log-rank *p* = 0.434). Among the 1338 patients with DM, there was no significant difference in the incidences of diabetic nephropathy between the *H. pylori*-positive and *H. pylori*-negative cases (52.7% vs. 52.0%, log-rank *p* = 0.798). Among 1338 patients with DM, there was no significant difference in the incidences of poor glycemic control between the *H. pylori*-positive and *H. pylori*-negative cases (78.5% vs. 80.9%, log-rank *p* = 0.292). A similar lack of significant differences was observed in the cohort that was diagnosed histologically (Figure 3).

### 3.3. Serological H. pylori Status and DM

Table 2 shows the risks of the various outcomes according to serologically determined *H. pylori* status. Among all subjects, *H. pylori*-positive status was not significantly associated with DM (HR: 0.98, 95% CI: 0.88–0.10; *p* = 0.763) or IGT (HR: 1.00, 95% CI: 0.96–1.05; *p* = 0.930). Furthermore, DM and IGT were consistently not associated with *H. pylori* status based on model 1 (age and sex), model 2 (model 1 plus BMI, blood pressure, drinking, smoking, and physical activity), or model 3 (model 2 plus triglycerides, HDL-cholesterol, and LDL-cholesterol) (*p* = 0.854 and *p* = 0.566).

Among the patients with DM, *H. pylori*-positive status was not significantly associated with diabetic nephropathy (HR: 0.99, 95% CI: 0.85–1.15; *p* = 0.875) or poor glycemic control (HR: 1.05, 95% CI: 0.93–1.18; *p* = 0.479). Furthermore, diabetic nephropathy and poor glycemic control were consistently not associated with *H. pylori* status after adjust for the potentially relevant covariates (*p* = 0.952 and *p* = 0.535).

### 3.4. Histological H. pylori Status and DM

Table 3 shows the risks of the various outcomes according to the histologically determined H. pylori status. Among all subjects, *H. pylori*-positive status was not significantly associated with DM (HR: 0.93, 95% CI: 0.84–1.03; *p* = 0.157), even after adjustment for the potentially confounding variables (HR: 0.93, 95% CI: 0.81–1.07; *p* = 0.311). However, *H. pylori*-positive status was significantly associated with IGT (HR: 0.94, 95% CI: 0.91–0.98; *p* = 0.003), and this significant association persisted after adjustment for the potentially relevant covariates (*p* = 0.002).

Among patients with DM, histologically determined *H. pylori*-positive status was not significantly associated with diabetic nephropathy (HR: 1.03, 95% CI: 0.89–1.19; *p* = 0.677) or poor glycemic control (HR: 0.99, 95% CI: 0.88–1.11; *p* = 0.854). The lack of significant associations persisted in the multivariable-adjusted analyses for diabetic nephropathy (*p* = 0.888) and poor glycemic control (*p* = 0.989).

## 4. Discussion

This large-scale two-cohort study revealed no significant associations between past *H. pylori* status and the development of DM, IGT, diabetic nephropathy, or poor glycemic control. This finding was further strengthened by the fact that the lack of significant associations held true for cases in which serological methods or histological methods were used to determine the *H. pylori* status. Previous studies have revealed mixed results regarding these relationships [12,13,14,15,16,17,18,19,22,23,24,29,30], which were likely related to discrepancies in study design, sample size, *H. pylori* detection method, DM-related testing and definition, and adjustment for potential confounders. In particular, most studies used a cross-sectional design, rather than a cohort design [12,13,15,16,22,23], or evaluated small samples of <1000 subjects [12,13,14,16,24,30]. Gunji et al. [12] evaluated 1107 subjects and revealed that, after adjusting for age, sex, BMI, waist girth, visceral and subcutaneous adipose tissue, smoking, alcohol, dietary habits, and physical activity, *H. pylori* infection was significantly associated with values from the homeostasis model assessment of insulin resistance (HOMA-IR). Chen et al. [15] evaluated 13,489 subjects from the National Health and Nutrition Examination Survey and, after adjusting for age, sex, smoking, education, ethnicity, and BMI, found a significant association between *H. pylori* status and HbA1c levels. However, despite their large samples and adjustment for potential confounders, both studies used cross-sectional designs.

Another important issue is the *H. pylori* detection method, with most studies only considering serological results. While this approach is useful for epidemiological studies, it may be less useful as a clinical diagnostic method, based on its low specificity. Other studies have also included patients with type 1 and type 2 DM, which have different pathogeneses [15,17], or have used different methods for identifying patients with DM [17,18,19,29]. Moreover, studies have failed to consider factors that might have influenced DM-related parameters, such as their treatment status, sociodemographic characteristics, and metabolic factors. Thus, the reported differences in HOMA-IR or HbA1c levels according to *H. pylori* status might have been related to unconsidered confounding factors, rather than *H. pylori* infection itself. The previous studies also evaluated insulin resistance based on HOMA-IR alone [12,13], despite the fact that insulin resistance is a cluster of physiological disorders that includes cytokine disturbances, subclinical inflammation, reactive oxygen species, and macrophage functions. Thus, more detailed information regarding insulin resistance and its parameters may be needed to understand the relationship with *H. pylori* infection. It is also important to consider that *H. pylori* infection might not induce a sufficiently strong inflammatory response to lead to the development of DM or worsening of glycemic control [29]. Therefore, while previous studies have suggested that *H. pylori* infection may lead to inflammation and DM, an alternate theory suggests that hyperglycemia may impair the host defense and predispose the patient to infection [31].

To the best of our knowledge, this is the first study to indicate that past *H. pylori* status was not associated with the development of type 2 DM or its complications in a large asymptomatic population, even after adjusting for potential confounding metabolic and lifestyle factors. The present study’s findings are also strengthened by the longitudinal analysis, rather than the use of a cross-sectional design, and the consistent lack of associations regardless of whether we evaluated serological or histological *H. pylori* status. These results are consistent with those from a similar study that evaluated a large sample of patients (*n* = 37,263) [22], and Lutsey et al. [23] failed to detect a significant association between DM and *H. pylori* status after adjusting for demographic factors. Recent studies have also failed to reveal clear evidence that *H. pylori* infection affects the host’s metabolic status [32,33]. Several studies have also evaluated whether eradication of *H. pylori* could influence glycemic control, with no effects observed in most studies [24,30,34,35,36], albeit with a conflicting result in another study [36]. Moreover, two retrospective cohort studies revealed that *H. pylori* eradication did not improve metabolic parameters, including HOMA-IR scores [24,30], which further suggests that *H. pylori* infection and DM may not be significantly related.

This study has several limitations. First, serological testing may be insufficient in cases without active *H. pylori* infection. However, we aimed to address this limitation by using histological findings, which provides better specificity, similar to other invasive testing methods, such as the rapid urease test. Second, we identified diabetic nephropathy based on albuminuria or a decreased eGFR, although these might have been caused by other renal diseases or hypertension. However, most patients with chronic illnesses are referred to outpatient clinics, rather than to health check-up centers, based on the South Korean healthcare insurance system. Third, the present study failed to consider various risk factors for insulin resistance or DM, including family history of DM, history of gestational DM, and polycystic ovary syndrome. Forth, only a single ethnic (Koreans) were included in this study. Research involving other ethnicities should proceed in the future.

In conclusion, even after adjusting for demographic and metabolic factors, this large cohort study failed to detect evidence that past *H. pylori* infection was associated with the development of DM, IGT, diabetic nephropathy, or poor glycemic control. Nevertheless, large prospective trials are needed to better assess the relationship between *H. pylori* infection and the development of DM.

## Figures and Tables

**Table 1:** Baseline characteristics of the study subjects according to *Helicobacter pylori* (*H. pylori*) status.

	All (*n* = 16091)	*H. pylori* (−) (*n* = 6700, 41.6%)	*H. pylori* (+) (*n* = 9391, 58.4%)	*p* Value
Age (year)	51.3	51.2 ± 7.6	51.4 ± 7.4	0.003
Sex (male, %)	60.2	59.1	61.0	0.015
Systolic BP (mmHg)	114.1	113.8 ± 15.2	114.3 ± 15.5	0.069
Diastolic BP (mmHg)	69.9	69.6 ± 10.2	70.0 ± 10.4	0.016
BMI (kg/m^2^)	23.8	23.8 ± 2.7	23.8 ± 2.7	0.309
Current smoker (%)	19.9	21.2	19.1	0.002
Heavy drinker (%)	14.9	14.9	14.9	0.917
Regular exercise (%)	44.8	45.5	44.3	0.146
Total cholesterol (mg/dl)	192.2	191.2 ± 31.7	192.9 ± 31.9	0.004
LDL-cholesterol (mg/dl)	127.7	126.1 ± 29.5	127.6 ± 29.6	<0.001
HDL-cholesterol (mg/dl)	57.3	57.9 ± 14.2	56.8 ± 13.9	<0.001
Triglycerides (mg/dl)	74.7	128.6 ± 81.0	127.0 ± 75.0	0.577
Fasting plasma glucose (mg/dl)	89.2	89.4 ± 9.5	89.1 ± 9.5	0.038
HbA1c (%)	5.3	5.3 ± 0.4	5.3 ± 0.4	0.346
Insulin (µIU/mL)	9.0	9.1 ± 3.9	9.0 ± 3.9	0.075
C-peptide (ng/mL)	1.8	1.9 ± 0.8	1.8 ± 0.7	<0.001
Urinary microalbumin (mg/g Cr)	2.6	2.6 ± 18.5	2.6 ± 8.8	0.443
Estimated GFR (mL/min/1.73 m^2^)	87.9	88.4 ± 12.2	87.6 ± 12.2	<0.001

BP, blood pressure; BMI, body mass index; LDL, low-density lipoprotein; HDL, high-density lipoprotein; HbA1c, glycated hemoglobin; GFR, glomerular filtration rate.

**Table 2:** Association between *Helicobacter pylori* (*H. pylori*) status and the risk of diabetes.

	Cases/n (%)	Unadjusted	Model 1	Model 2	Model 3
		HR (95% CI)	HR (95% CI)	HR (95% CI)	HR (95% CI)
DM					
*H. pylori* (−)	569/6700 (8.5)	1	1	1	1
*H. pylori* (+)	769/9391 (8.2)	0.98 (0.88–0.10)	0.97 (0.87–1.08)	1.01 (0.89–1.16)	1.01 (0.88–1.16)
*P* value		0.763	0.578	0.863	0.854
IGT					
*H. pylori* (−)	3460/6700 (51.6)	1	1	1	1
*H. pylori* (+)	4791/9391 (51.0)	1.00 (0.96–1.05)	0.98 (0.94–1.03)	0.99 (0.94-1.04)	0.98 (0.93–1.04)
*P* value		0.930	0.476	0.628	0.566
DM nephropathy					
*H. pylori* (−)	300/569 (52.7)	1	1	1	1
*H. pylori* (+)	400/769 (52.0)	0.99 (0.85–1.15)	0.99 (0.85–1.15)	1.00 (0.83–1.21)	0.99 (0.82–1.21)
*P* value		0.875	0.871	0.990	0.952
Poor DM control					
*H. pylori* (−)	446/568 (78.5)	1	1	1	1
*H. pylori* (+)	621/768 (80.9)	1.05 (0.93–1.18)	1.06 (0.94–1.20)	1.06 (0.91–1.23)	1.05 (0.90–1.22)
*P* value		0.479	0.352	0.465	0.535

DM, diabetes mellitus; IGT, impaired glucose tolerance; HR, hazard ratio; CI, confidence interval; Model 1: adjusted for age, sex; Model 2: adjusted for variables in model 1, plus body mass index; systolic and diastolic blood pressure, drinking, smoking, and physical activity. Model 3: adjusted for variables in model 2, plus triglyceride, HDL-cholesterol, and LDL-cholesterol.

**Table 3:** Association between *Helicobacter pylori* (*H. pylori*) status and the risk of diabetes.

	Cases/n (%)	Unadjusted	Model 1	Model 2	Model 3
		HR (95% CI)	HR (95% CI)	HR (95% CI)	HR (95% CI)
DM					
*H. pylori* (−)	2578/36283 (7.1)	1	1	1	1
*H. pylori* (+)	453/6068 (7.5)	0.93 (0.84–1.03)	0.94 (0.85–1.04)	0.93 (0.82–1.07)	0.93 (0.81–1.07)
*P* value		0.157	0.247	0.313	0.311
IGT					
*H. pylori* (−)	19075/36283 (52.6)	1	1	1	1
*H. pylori* (+)	3219/6068 (53.0)	0.94 (0.91–0.98)	0.95 (0.91–0.98)	0.93 (0.89–0.98)	0.93 (0.89–0.97)
*P* value		0.003	0.005	0.003	0.002
DM nephropathy					
*H. pylori* (−)	1172/2578 (45.5)	1	1	1	1
*H. pylori* (+)	212/452 (46.9)	1.03 (0.89–1.19)	1.03 (0.89–1.19)	0.97 (0.80–1.18)	0.99 (0.81–1.20)
*P* value		0.677	0.701	0.772	0.888
Poor DM control					
*H. pylori* (−)	2020/2578 (78.4)	1	1	1	1
*H. pylori* (+)	352/453 (77.7)	0.99 (0.88–1.11)	1.00 (0.90–1.13)	1.01 (0.86–1.18)	1.00 (0.86–1.17)
*P* value		0.854	0.940	0.947	0.989

DM, diabetes mellitus; IGT, impaired glucose tolerance; HR, hazard ratio; CI, confidence interval; Model 1: adjusted for age, sex; Model 2: adjusted for variables in model 1, plus body mass index; systolic and diastolic blood pressure, drinking, smoking, and physical activity Model 3: adjusted for variables in model 2, plus triglyceride, HDL-cholesterol, and LDL-cholesterol.
